# Vasoprotection by heme oxygenase-1: interactions with soluble guanylate cyclase

**DOI:** 10.1186/1471-2210-11-S1-O32

**Published:** 2011-08-01

**Authors:** William Durante

**Affiliations:** 1 Department of Medical Pharmacology and Physiology, University of Missouri, Columbia, Missouri, 65212, USA

## Background

Heme oxygenase-1 (HO-1) is a highly inducible enzyme that metabolizes heme to generate equimolar amounts of carbon monoxide (CO), biliverdin, and ferrous iron. Subsequently, biliverdin is metabolized to the potent antioxidant bilirubin by biliverdin reductase. Compelling experimental evidence indicates that HO-1 and its end products protect against the development of vascular dysfunction in animal models atherosclerosis, post-angioplasty restenosis, vein graft stenosis, thrombosis, myocardial infarction, and hypertension.

## Results

Studies from our laboratory and others have identified HO-1 as a critical regulator of vascular remodeling following arterial injury. Pharmacological induction or gene delivery of HO-1 suppresses neointima formation following arterial injury while HO-1 deletion exacerbates lesion development. Similarly, local or systemic administration of CO inhibits intimal thickening following arterial injury. Studies in cultured vascular smooth muscle cells (SMCs) reveal that overexpression of HO-1 or exogenously applied CO blocks SMC proliferation. The anti-proliferative action of CO is dependent on the activation of soluble guanylate cyclase and is associated with the arrest of SMCs in the G0/G1 phase of the cell cycle. Interestingly, the soluble guanylate cyclase stimulator YC-1 potentiates the anti-proliferative actions of CO in cultured SMCs and uniquely stimulates the production of CO by inducing HO-1 gene expression, providing a novel mechanism by which this agent is able to amplify cGMP production. YC-1 also suppresses neointima formation, and this is associated with an increase in cGMP levels in injured arteries. Finally, HO-1 deficiency impairs endothelium-dependent vasorelaxation and alters vasorelaxation responses to soluble guanylate cyclase stimulators and activators. The alteration in vasoreactivity in HO-1-deficient animals is accompanied by a pronounced decline in soluble guanylate cyclase expression.

## Conclusion

Collectively, these findings demonstrate that HO-1 elicits important protective actions in the vasculature by stimulating soluble guanylate cyclase activity and preserving soluble guanylate cyclase expression.

